# Co-expression network analysis identified six hub genes in association with metastasis risk and prognosis in hepatocellular carcinoma

**DOI:** 10.18632/oncotarget.16896

**Published:** 2017-04-06

**Authors:** Pengfei Chen, Fan Wang, Juerong Feng, Rui Zhou, Ying Chang, Jing Liu, Qiu Zhao

**Affiliations:** ^1^ Department of Gastroenterology, Zhongnan Hospital of Wuhan University, Wuhan, China; ^2^ Hubei Clinical Center and Key Laboratory of Intestinal and Colorectal Diseases, Wuhan, China; ^3^ Department of Gastroenterology, The Central Hospital of Enshi Autonomous Prefecture, Enshi, China

**Keywords:** hepatocellular carcinoma, co-expression network analysis, hub genes, metastasis risk, prognosis

## Abstract

Hepatocellular carcinoma (HCC) has a high incidence and mortality worldwide, and its carcinogenesis and progression are influenced by a complex network of gene interactions. A weighted gene co-expression network was constructed to identify gene modules associated with the clinical traits in HCC (*n* = 214). Among the 13 modules, high correlation was only found between the red module and metastasis risk (classified by the HCC metastasis gene signature) (R^2^ = −0.74). Moreover, in the red module, 34 network hub genes for metastasis risk were identified, six of which (ABAT, AGXT, ALDH6A1, CYP4A11, DAO and EHHADH) were also hub nodes in the protein-protein interaction network of the module genes. Thus, a total of six hub genes were identified. In validation, all hub genes showed a negative correlation with the four-stage HCC progression (*P* for trend < 0.05) in the test set. Furthermore, in the training set, HCC samples with any hub gene lowly expressed demonstrated a higher recurrence rate and poorer survival rate (hazard ratios with 95% confidence intervals > 1). RNA-sequencing data of 142 HCC samples showed consistent results in the prognosis. Gene set enrichment analysis (GSEA) demonstrated that in the samples with any hub gene highly expressed, a total of 24 functional gene sets were enriched, most of which focused on amino acid metabolism and oxidation. In conclusion, co-expression network analysis identified six hub genes in association with HCC metastasis risk and prognosis, which might improve the prognosis by influencing amino acid metabolism and oxidation.

## INTRODUCTION

Hepatocellular carcinoma (HCC) is one of the most common malignancies worldwide, and it is the second leading cause of cancer-related death among males [1]. Multiple factors were reported to be related with the carcinogenesis and progression in HCC, like chronic infection of hepatitis B virus (HBV) or hepatitis C virus (HCV), alcohol consumption and smoking [2]. However, the mechanism remains obscure. In recent years, with the development of gene microarray and RNA sequencing, gene expression profiling has been used to identify genes associated the carcinogenesis and development of HCC. Through gene ontology analysis, the mechanism has been partially illustrated. However, most studies focused on the screening of differentially expressed genes, and ignored the high interconnection between genes although genes with similar expression patterns are probably correlated in function [3]. In this study, we adopted the systems biology-based approach of weighted gene co-expression network analysis (WGCNA) to construct a co-expression network based on the relationship between genes, and identified significant gene modules and hub genes associated with the clinical traits in HCC.

## RESULTS

### DEGs screening

After outlier exclusion, the gene profiles of 443 samples were analyzed. Under the threshold of FDA < 0.05 and |log_2_FC| > 0.585, a total of 3670 DEGs (2213 up-regulated and 1457 down-regulated in HCC) were selected for subsequent analysis.

### Characteristics of the included samples in co-expression analysis

**Figure 1:**
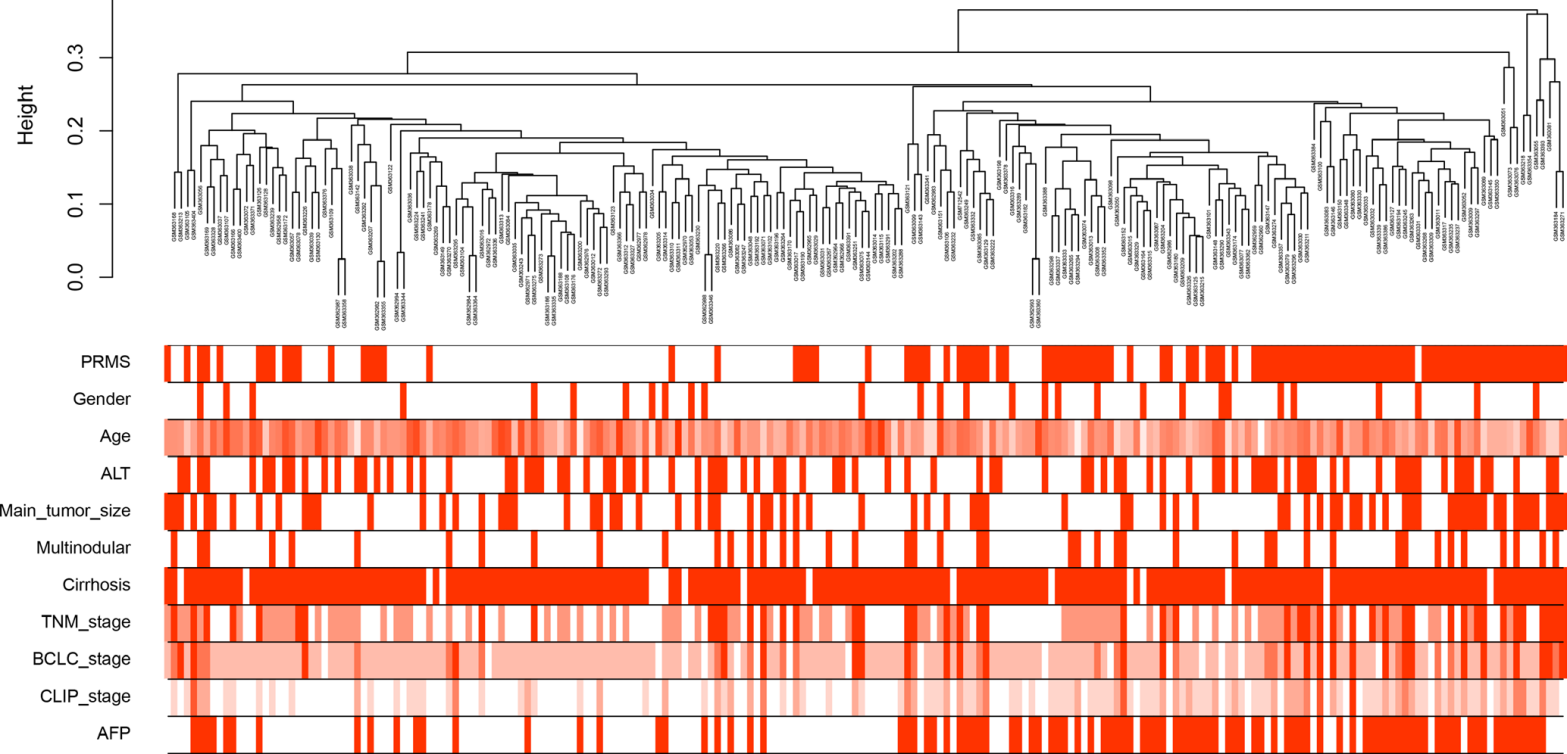
Clustering dendrogram of 214 tumor samples and the clinical traits The clustering was based on the expression data of differentially expressed genes between tumor samples and non-tumor samples in hepatocellular carcinoma. PRMS: predicted risk metastasis signature. ALT: alanine aminotransferase. TNM: Tumor Node Metastasis. BCLC: Barcelona Clinic Liver Cancer. CLIP: Cancer of the Liver Italian Program. AFP: alpha-fetoprotein. The red color represented high metastasis risk, female, high ALT levels (> 50 U/L), large tumor size (> 5 cm), multinodular, cirrhosis and high AFP levels (> 300 ng/ml). The color intensity was proportional to older age and higher stage of TNM, BCLC and CLIP.

214 HCC samples with complete clinical data were included in co-expression analysis. The samples had an average age of 50.7 years, and a high proportion of males (86.4%, 185/214) (Figure 1). 87 (40.7%) cases had high levels of serum alanine aminotransferase (ALT, > 50 U/L), and 97 (45.3%) with high levels of serum alpha-fetoprotein (AFP, > 300 ng/ml). 197 (92.1%) cases were concomitant with cirrhosis. 44 (20.6%) cases were multinodular, and the main tumor size in 77 (36.0%) cases were more than 5 cm. Three kinds of tumor staging were adopted: TNM (Tumor Node Metastasis), BCLC (Barcelona Clinic Liver Cancer) and CLIP (Cancer of the Liver Italian Program). Metastasis risk (PRMS) was predicted based on the 161 gene HCC metastasis signature, and 103 (48.1%) cases were at high risk [4].

### Co-expression network construction and key modules identification

**Figure 2:**
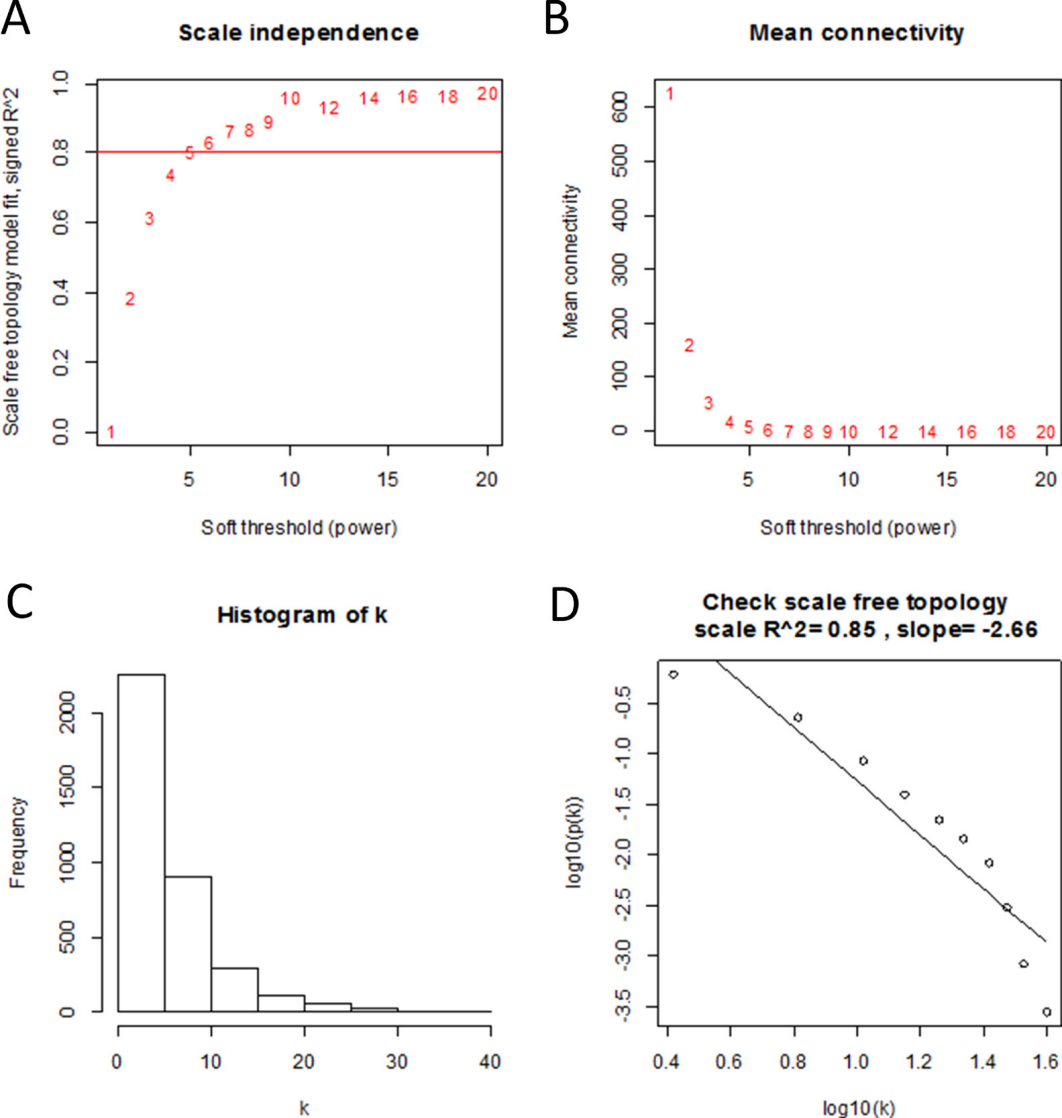
Determination of soft-thresholding power in the weighted gene co-expression network analysis (WGCNA) (**A**) Analysis of the scale-free fit index for various soft-thresholding powers (β). (**B**) Analysis of the mean connectivity for various soft-thresholding powers. (**C**) Histogram of connectivity distribution when β = 6. (**D**) Checking the scale free topology when β = 6.

**Figure 3:**
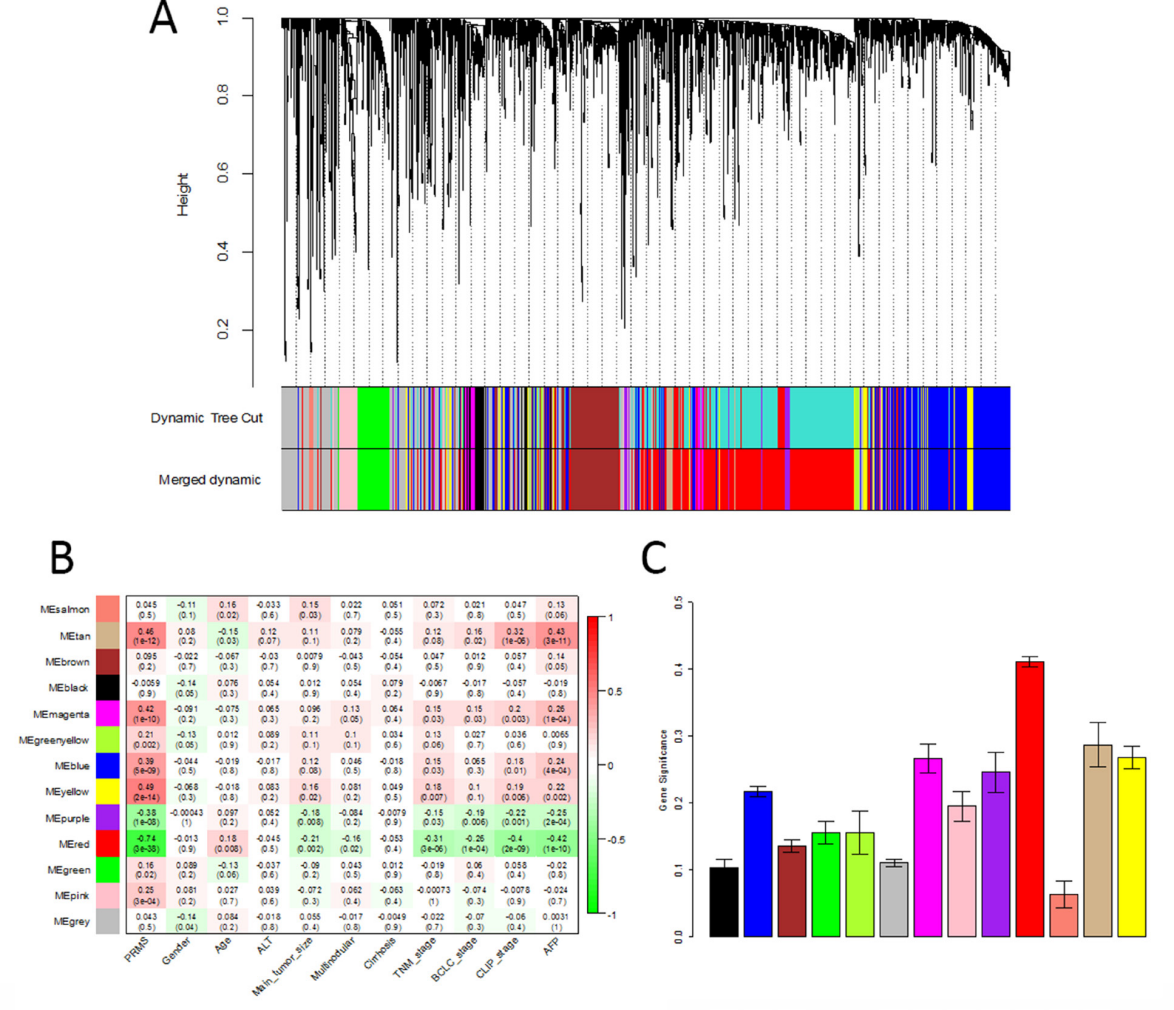
Identification of modules associated with clinical traits (**A**) Clustering dendrogram of all differentially expressed genes in 214 samples of hepatocellular carcinoma. (**B**) Heatmap between the correlation between module eigengenes and clinical traits. Each cell contained the corresponding correlation and *P value* (**C**) Distribution of average gene significance and errors in the modules associated with the metastasis risk in hepatocellular carcinoma.

Using “WGCNA” package in R, the DEGs with similar expression patterns were grouped into modules via the average linkage hierarchical clustering. Here, the power of β = 6 (scale free R^2^ = 0.85) was selected as the soft-thresholding to ensure a scale-free network (Figure 2). A total of 13 modules were identified (Figure 3A). Two methods were used to test the relevance between each module and clinical traits. Firstly, the ME in several modules showed a correlation with certain clinical traits (*P <* 0.05). However, most of the correlations were low to moderate (R^2^ < 0.5), and only the correlation between the red module and metastasis risk (PRMS) was high (*P* = 3 × 10^−38^, R^2^ = −0.74) (Figure 3B). Secondly, in relation with metastasis risk, the red module also had the highest MS (Figure 3C). Thus, the red module with metastasis risk was identified as the clinical significant module, which was extracted for further analysis.

### Identification of hub genes for metastasis risk in the red module

**Figure 4:**
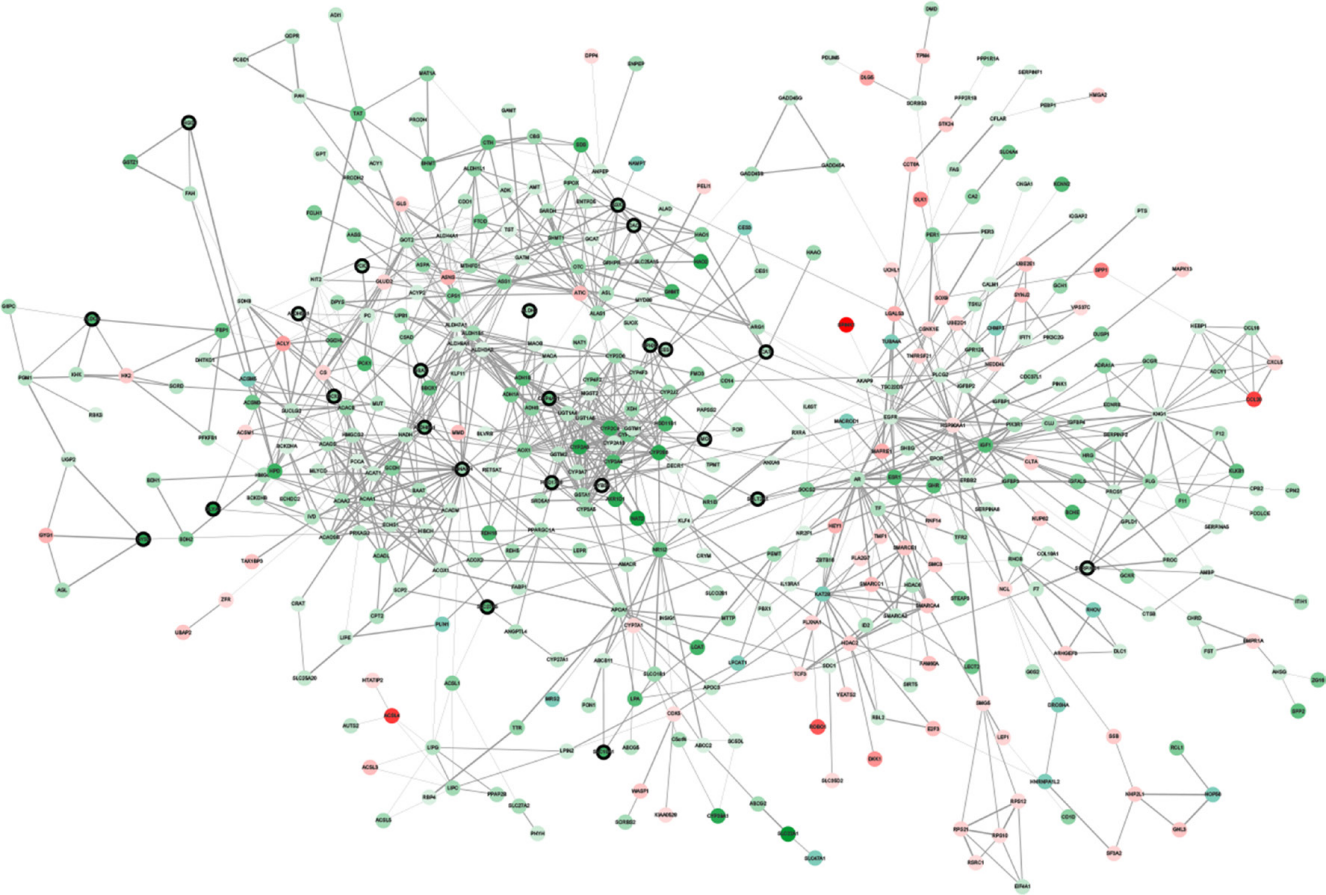
Protein–protein interaction network of genes in the red module The color intensity in each node was proportional to change fold of expression in comparison to non-tumor samples (up-regulation in red and down-regulation in green). The nodes with bold circle represented the hub genes identified by co-expression network analysis. The edge width was proportional to the score of protein-protein interaction based on the STRING database.

Highly connected hub genes in a module play important roles in the biological processes [5]. Therefore, 34 genes with the high connectivity (weighted correlation coefficients > 0.8) in red module were taken as candidate hub genes for metastasis risk in the module (Table 1). Furthermore, We also constructed a network of protein-protein interaction (PPI) for the genes in red module according to the STRING database, and six hub genes (ABAT, AGXT, ALDH6A1, CYP4A11, DAO) in the co-expression network were also identified as hub nodes in the PPI network (Figure 4). Finally, these six genes were regarded as “real” hub genes for metastasis risk and selected for further analyses.

**Table 1:** Hub genes in the module related with metastasis risk

Gene	Probe	Co-expression analysis			Hub gene inPPI network	DEG analysis	
		p.Weighted	q.Weighted	cor.Weighted		logFC	FDR
ABAT	209459_s_at	0	0	−0.85	YES	−1.80	8.77E-54
AGXT	210326_at	0	0	−0.80	YES	−1.88	5E-38
ALDH6A1	221590_s_at	0	0	−0.83	YES	−2.26	1.98E-63
CYP4A11	211231_x_at	0	0	−0.81	YES	−2.53	3.28E-86
DAO	206878_at	0	0	−0.83	YES	−1.38	2.75E-33
EHHADH	205222_at	0	0	−0.81	YES	−1.55	7.15E-45
ABCA6	217504_at	0	0	−0.80	NO	−1.55	6.37E-42
AGXT2L1	221008_s_at	0	0	−0.82	NO	−2.53	5.85E-44
ALDH2	201425_at	0	0	−0.85	NO	−1.50	4.39E-68
ALDH5A1	203608_at	0	0	−0.81	NO	−1.03	9.4E-29
ALDOB	204704_s_at	0	0	−0.83	NO	−2.90	4.04E-57
APOC4	206738_at	0	0	−0.85	NO	−2.07	2.73E-40
CAT	201432_at	0	0	−0.84	NO	−1.18	2.23E-49
CES2	213509_x_at	0	0	−0.83	NO	−1.41	9.92E-31
CYB5A	207843_x_at	0	0	−0.80	NO	−0.72	1.26E-21
DCXR	217973_at	0	0	−0.88	NO	−1.87	4.93E-53
DHRS1	213279_at	0	0	−0.83	NO	−1.73	6.04E-59
EPHX2	209368_at	0	0	−0.82	NO	−1.95	1.06E-59
F13B	207810_at	0	0	−0.81	NO	−0.85	2.7E-11
FMO4	206263_at	0	0	−0.83	NO	−1.26	8.51E-32
GLYAT	222083_at	0	0	−0.82	NO	−3.57	1.87E-90
GYS2	214621_at	0	0	−0.81	NO	−3.42	1.93E-81
HAGH	205012_s_at	0	0	−0.88	NO	−1.29	2.92E-43
HGD	205221_at	0	0	−0.80	NO	−1.59	4.94E-40
HRSP12	203790_s_at	0	0	−0.81	NO	−1.48	1.18E-43
HSD17B6	37512_at	0	0	−0.81	NO	−1.87	7.7E-30
PCK2	202847_at	0	0	−0.87	NO	−1.53	3.88E-49
PXMP2	219076_s_at	0	0	−0.82	NO	−1.08	1.48E-32
RGN	210751_s_at	0	0	−0.81	NO	−1.47	3.99E-51
SEC14L2	204541_at	0	0	−0.87	NO	−1.78	2.27E-53
SERPINC1	210049_at	0	0	−0.82	NO	−1.09	1.34E-16
SLC10A1	207185_at	0	0	−0.85	NO	−3.04	2.13E-50
SLC27A5	219733_s_at	0	0	−0.90	NO	−2.62	3.86E-66
SULT2A1	206292_s_at	0	0	−0.85	NO	−1.77	1.62E-28

Abbreviations: PPI: protein-protein interaction. FC: fold change. DEG: differentially expressed genes. FDR: false discovery rate.

### Hub genes validation

The hub genes were identified in strong correlation with HCC metastasis risk. As the metastasis risk increased with HCC progression and high metastasis risk indicated a poor prognosis, we validated the hub genes indirectly by investigating their roles in HCC progression and prognosis.

**Figure 5:**
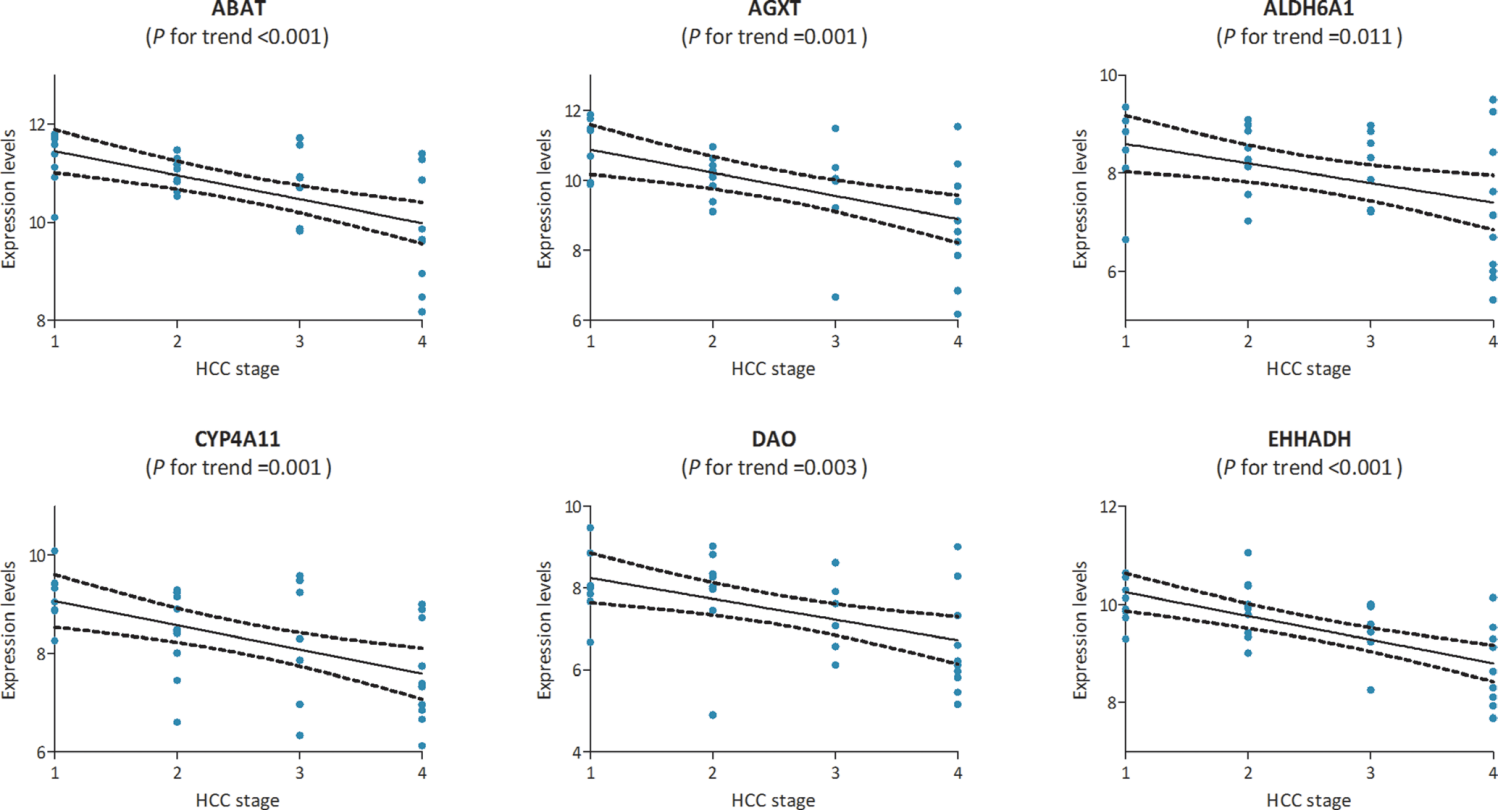
The correlation between the expression levels of ABAT, AGXT, ALDH6A1, CYP4A11, DAO and EHHADH and the disease progression of hepatocellular carcinoma (HCC) (based on microarray data of GSE6764) Stage: 1 for very early HCC, 2 for early HCC, 3 for advanced HCC, and 4 for very advanced HCC.

**Figure 6:**
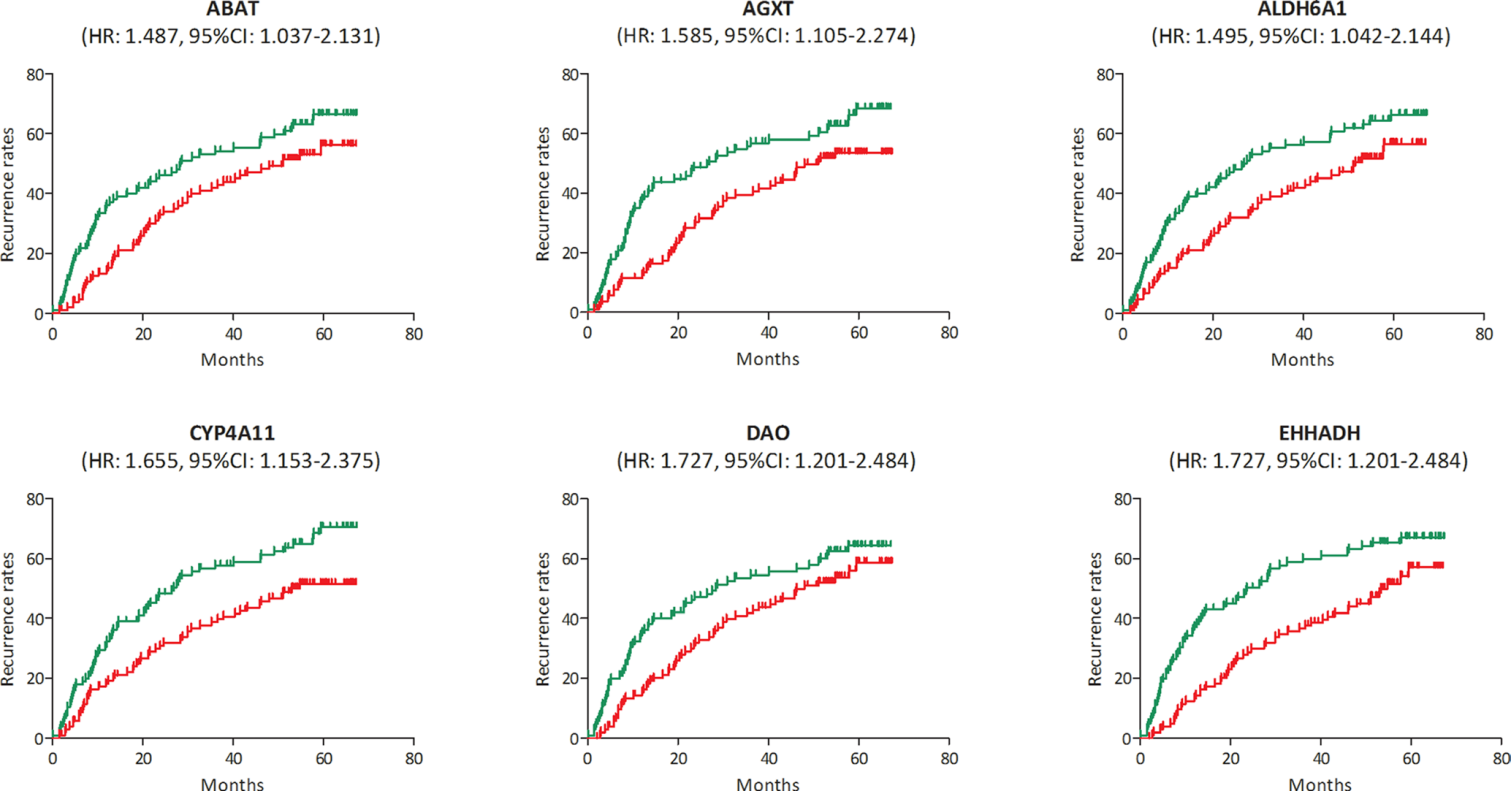
Recurrence analysis of the association between the expression levels of ABAT, AGXT, ALDH6A1, CYP4A11, DAO and EHHADH and recurrence rates in hepatocellular carcinoma (HCC) (based on microarray data of GSE14520) Red line represented the samples with gene highly expressed, and green line was for the samples with gene lowly expressed. HR: hazard ratio, CI: confidence interval.

**Figure 7:**
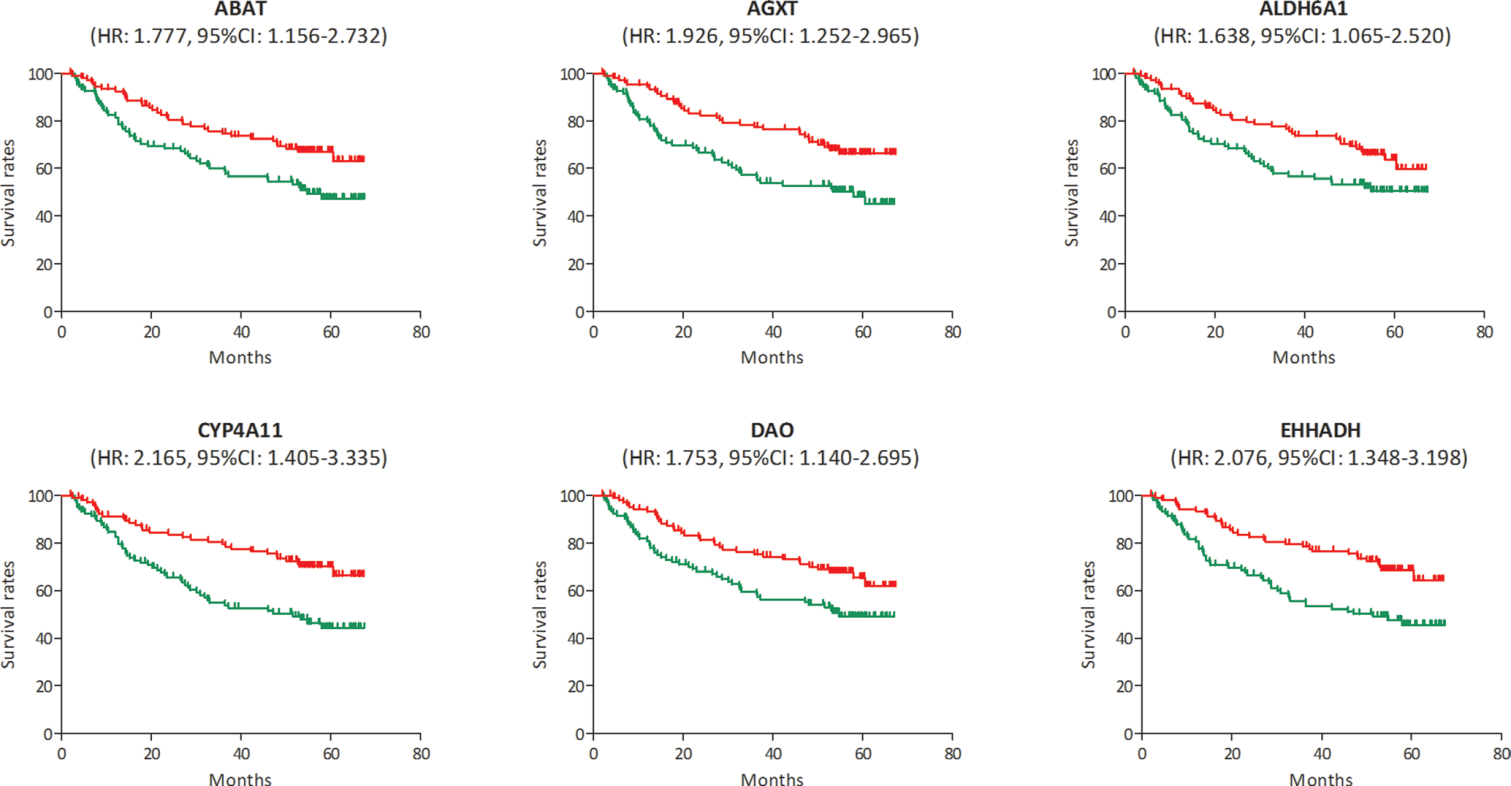
Survival analysis of the association between the expression levels of ABAT, AGXT, ALDH6A1, CYP4A11, DAO and EHHADH and survival rates in hepatocellular carcinoma (HCC) (based on microarray data of GSE14520) Red line represented the samples with gene highly expressed, and green line was for the samples with gene lowly expressed. HR: hazard ratio, CI: confidence interval.

In the test set of GSE6764, linear regression analyses were conducted on the six hub genes, all of which showed a negative correlation with HCC progression (*P* for trend < 0.05) (Figure 5). In the training set, based on the microarray data of 214 HCC samples, we investigated the role of hub genes in HCC prognosis. All samples were divided into two groups according to the expression levels of hub genes respectively. We found a higher recurrence rate and a poorer survival rate in the samples with any hub gene lowly expressed (Figures 6, 7). In recurrence analysis, the hazard ratios (HR) and corresponding 95% confidence intervals (CI) were 1.487 (1.037–2.131) for ABAT, 1.585 (1.105–2.274) for AGXT, 1.495 (1.042–2.144) for ALDH6A1, 1.655 (1.153–2.375) for CYP4A11, 1.727 (1.201–2.484) for DAO and 1.727 (1.201–2.484). In survival analysis, the HRs and CIs were 1.777 (1.156–2.732) for ABAT, 1.926 (1.252–2.965) for AGXT, 1.638 (1.065–2.520) for ALDH6A1, 2.165 (1.405–3.335) for CYP4A11, 1.753 (1.140–2.695) for DAO and 2.076 (1.348–3.198) for EHHADH.

**Figure 8:**
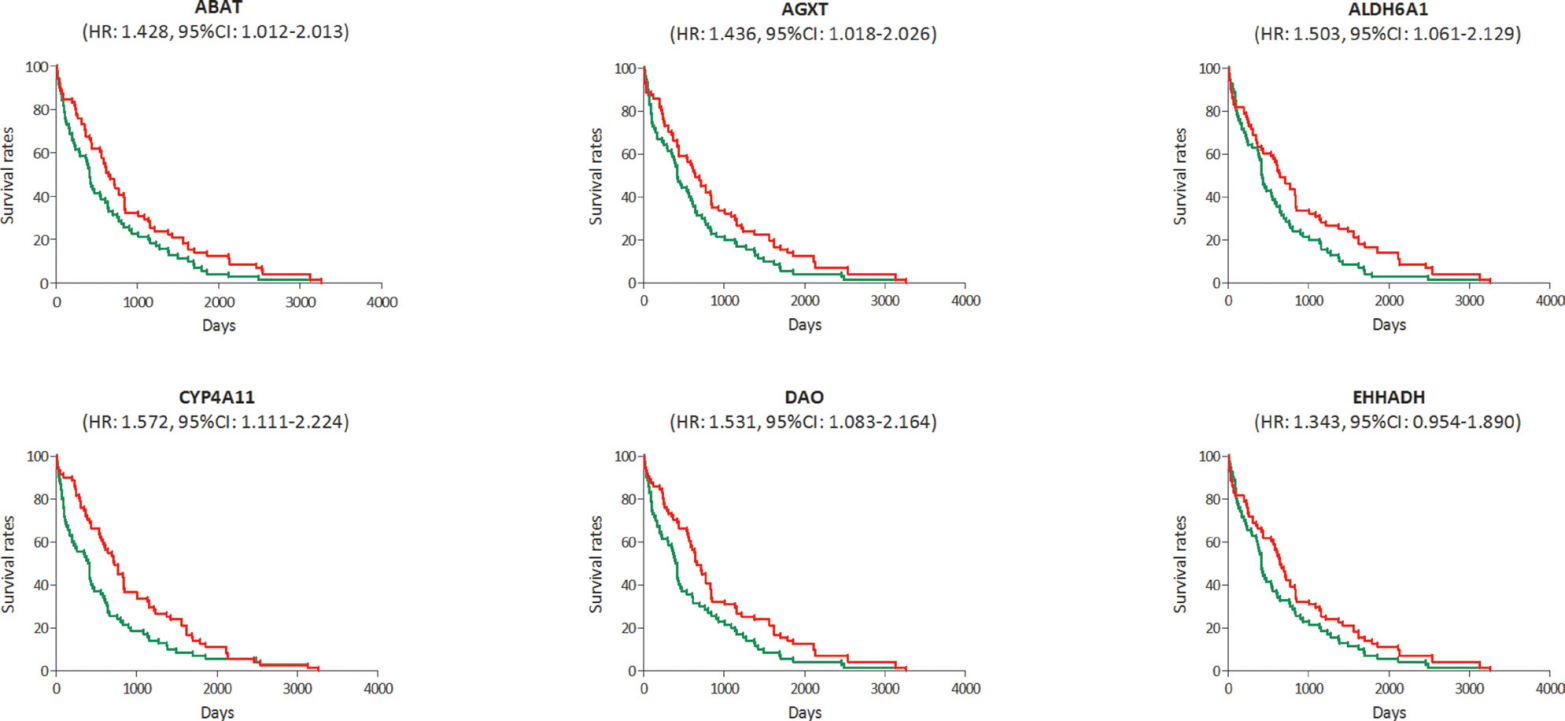
Survival analysis of the association between the expression levels of ABAT, AGXT, ALDH6A1, CYP4A11, DAO and EHHADH and survival rates in hepatocellular carcinoma (HCC) (based on RNA-sequencing data) Red line represented the samples with gene highly expressed, and green line was for the samples with gene lowly expressed. HR: hazard ratio, CI: confidence interval.

In the RNA-sequencing data of 423 HCC samples, the survival data in 142 samples were available. We also found a poorer survival rate in the samples with low expression levels of ABAT, AGXT, ALDH6A1, CYP4A11 or DAO, and it showed a deadline effect in EHHADH (Figure 8). The HRs and CIs were 1.428 (1.012–2.013) for ABAT, 1.436 (1.018–2.026) for AGXT, 1.503 (1.061–2.129) for ALDH6A1, 1.572 (1.111–2.224) for CYP4A11, 1.531 (1.083–2.164) for DAO and 1.343 (0.954–1.890) for EHHADH.

### Gene set enrichment analysis

**Figure 9:**
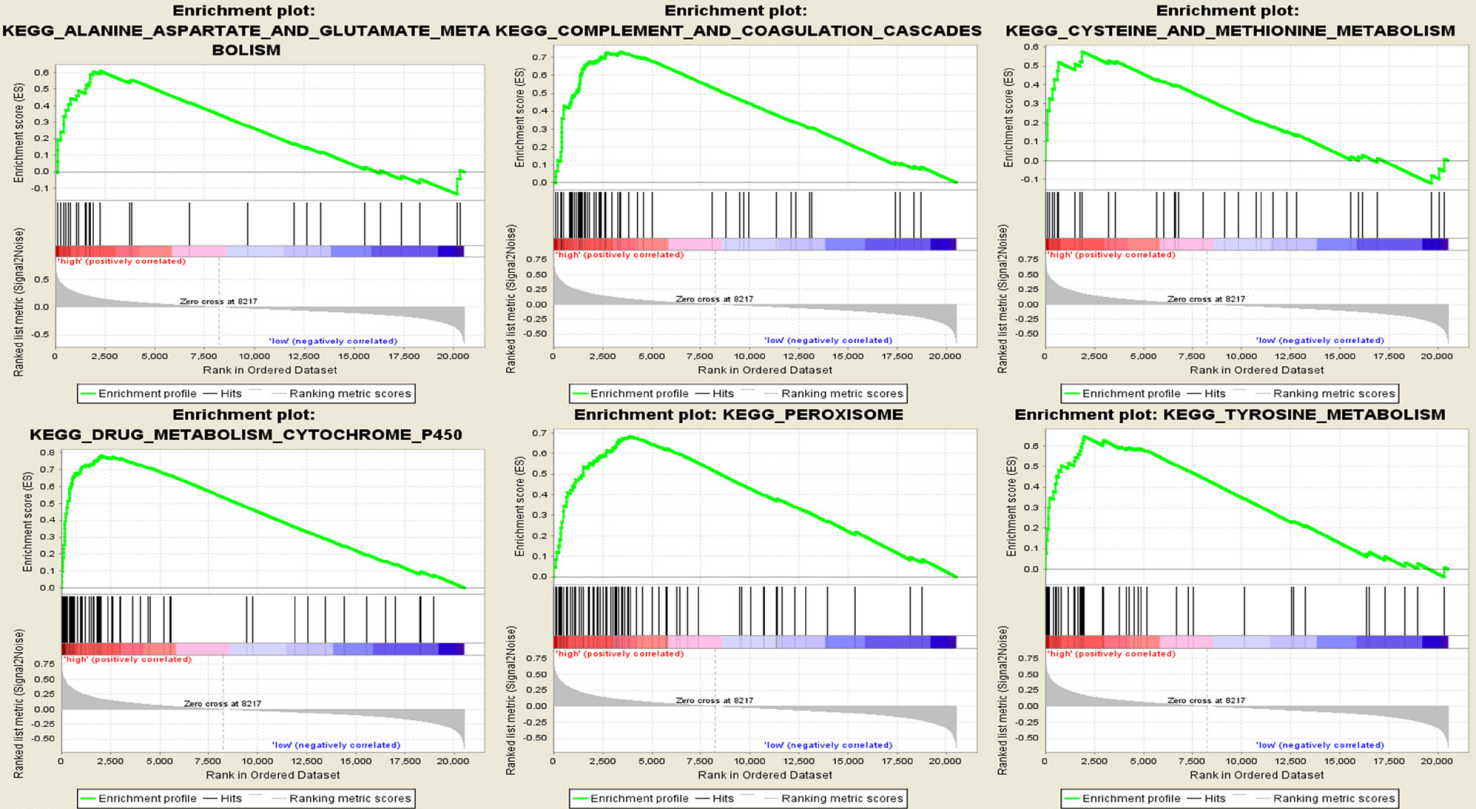
Gene set enrichment analysis (GSEA) Only listed the six most common functional gene sets enriched in hepatocellular carcinoma samples with CYP4A11 highly expressed.

To identify potential function of the hub genes, GSEA was conducted respectively to search KEGG (Kyoto Encyclopedia of Genes and Genomes) pathways enriched in the samples with the gene highly expressed. A total of 24 functional gene sets were enriched in the samples with high expression levels of any hub gene, and most sets focused on amino acid metabolism and oxidation (Supplementary Table 1). Six gene sets were enriched in the samples with at least three hub genes highly expressed, namely “alanine, aspartate and glutamate metabolism”, “complement and coagulation cascades”, “cysteine and methionine metabolism”, “drug metabolism cytochrome P450”, “peroxisome” and “tyrosine metabolism” (Figure 9).

## DISCUSSION

Hepatocellular carcinoma is the most frequent malignant tumor in the liver, and its mortality is high which contributes to the high frequency of late-stage disease, metastasis and the “field effect” [6]. Currently, surgery is the most effective treatment, but the recurrence rate is high. As metastasis contributes to 90% of all cancer related death, it is important for metastasis risk prediction [7]. For those with high risk of metastasis, additional therapies were needed.

In the study of Roessler et al. and their previous study, they identified a 161 gene signature as a metastasis risk classifier, which was also validated in another cohort [6, 8]. The signature could also predict HCC survival successfully in the cases with early disease and solitary tumors. Furthermore, it predicted particularly well for early recurrence risk. In our study, we also identified six genes (ABAT, AGXT, ALDH6A1, CYP4A11, DAO and EHHADH) in association with the high risk in metastasis prediction, and none of the genes were included in the 161-gene set of Roessler et al. In further analyses, these six genes were also significantly associated with HCC prognosis including recurrence and survival. RNA sequencing data showed consistent results, indicating our results were stable and independent of cohorts and gene profiling technologies.

ABAT (4-aminobutyrate aminotransferase) is responsible for catabolism of γ- aminobutyric acid (GABA) (an important and mostly inhibitory neurotransmitter in the central nervous system) into succinic semialdehyde [9]. Reis et al. found that ABAT was a protein biomarker with high sensitivity (84.4%/84.4%) in the diagnosis of hepatocellular differentiation and hepatoid adenocarcinomas [10].

AGXT (alanine-glyoxylate aminotransferase) is expressed only in the liver and the encoded protein is localized mostly in the peroxisomes, where it is involved in glyoxylate detoxification [11]. In Kjersem et al. study, AGXT polymorphisms was associated with clinical outcome in metastatic colorectal cancer patients with 5-fluorouracil/oxaliplatin [12].

ALDH6A1 (aldehyde dehydrogenase 6 family, member A1) encodes a member of the aldehyde dehydrogenase protein family, and the encoded protein is a mitochondrial methylmalonate semialdehyde dehydrogenase that plays a role in the valine and pyrimidine catabolic pathways [13]. Liu et al. also found that ALDH6A1 was down-regulated in HCC [14].

CYP4A11 (cytochrome P450, family 4, subfamily A, polypeptide 11) encodes a member of the cytochrome P450 superfamily of enzymes, which are monooxygenases and catalyze many reactions involved in drug metabolism and synthesis of cholesterol, steroids and other lipids [5]. Wnt/β-catenin signaling was abnormally activated in the progression of HCC, and activation of the Wnt/β-catenin pathway could prevent peroxisome proliferator-activated receptor (PPAR) α-mediated induction of CYP4A11 [15, 16].

DAO (D-amino-acid oxidase) encodes the peroxisomal enzyme D-amino acid oxidase, which is a flavoprotein that uses flavin adenine dinucleotide (FAD) as its prosthetic group [17]. Fang et al. found that tumor-targeted delivery of polyethylene glycol (PEG)-conjugated DAO produced remarkable antitumor activity via enzymatic generation of hydrogen peroxide (H_2_O_2_) [18].

EHHADH (enoyl-CoA hydratase and 3-hydroxyacyl CoA dehydrogenase) encodes a bifunctional enzyme which is one of the four enzymes of the peroxisomal β-oxidation pathway [19]. Suto et al. also found decreased expression of EHHADH in HCC [20].

The six hub genes showed a protective role in carcinogenesis mainly by correlating with the amino acid metabolism and oxidation. In gene set enrichment analysis, we also found that the gene sets associated with amino acid metabolism and oxidation were enriched in the samples with hub genes highly expressed.

In conclusion, co-expression network analysis identified six hub genes in association with HCC metastasis risk and prognosis, which might improve the prognosis by influencing amino acid metabolism and oxidation.

## MATERIALS AND METHODS

### Data collection

Normalized data of gene expression and related clinical data were downloaded from Gene Expression Omnibus (GEO) database (http://www.ncbi.nlm.nih.gov/geo/). Dataset GSE14520 was used as a training set to construct expression network and identify hub genes in this study. This dataset was based on the microarray platform of Affymetrix HT Human Genome U133A Array (HT_HG-U133A), and included 225 samples of hepatocellular carcinoma (HCC) and 220 samples of non-tumor tissues. Another independent dataset of GSE6764 was downloaded from GEO database and used as a test set to verify our results. This dataset was based on the platform of Affymetrix Human Genome U133 Plus 2.0 Array (HG-U133_Plus_2) and included 35 HCC samples covering four stepwise pathological stages of HCC progression (including very early HCC, early HCC, advanced HCC and very advanced HCC). Moreover, RNA-sequencing data of 423 HCC samples were also downloaded from The Cancer Genome Atlas (TCGA) database (https://genome-cancer.ucsc.edu/) to further verify our results. The gene expression data were based on the RNA-sequencing technology of IlluminaHiseq.

### Data preprocessing

Microarray quality was assessed by sample clustering according to the distance between different samples in Pearson's correlation matrices, and a height cut of 0.2 was chosen to identify potential microarray outliers. Two samples (GSM363045 and GSM363217) were detected as outliers and removed from the subsequent analysis (Supplementary Figure 1).

### Differentially expressed genes (DEGs) screening

The “limma” (linear models for microarray data) R package was used to screen the DEGs between HCC tumor tissues and non-tumor tissues. The false discovery rate (FDA) < 0.05 and |log2 fold change (FC)| > 0.585 were chosen as the cut-off criteria.

### Co-expression network construction

The “WGCNA” package in R was used to construct co-expression network for the DEGs in 214 tumor samples (one was excluded for outlier and ten were for the absence of clinical data) [21]. At first, the Pearson's correlation matrices were calculated for all pair-wise genes. Then, a weighted adjacency matrix was constructed using a power function a_mn_=|c_mn_|^β^ (c_mn_=Pearson's correlation between gene m and gene n; a_mn_=adjacency between gene m and gene n). β was a soft-thresholding parameter that could emphasize strong correlations between genes and penalize weak correlations. Next, the adjacency was transformed into topological overlap matrix (TOM), which could measure the network connectivity of a gene defined as the sum of its adjacency with all other genes for network generation [22]. To classify genes with similar expression profiles into gene modules, average linkage hierarchical clustering was conducted according to the TOM-based dissimilarity measure with a minimum size (gene group) of 20 for the resulted dendrogram [23].

### Identification of clinical significant modules

Two approaches were used to identify modules related with clinical traits. First, module eigengenes (MEs) were defined as the first principal component in the principal component analysis for each gene module, which could summarize the expression patterns of all genes into a single characteristic expression profile within a given module. Thus, we calculated the correlation between MEs and clinical traits to identify the most relevant module. Second, gene significance (GS) was defined as the log10 transformation of the *P value* in the linear regression between gene expression and clinical traits, and module significance (MS) was defined as the average GS for all the genes in a module. The module with the maximal absolute MS among all the selected modules was usually considered as the one related with clinical trait. Finally, the module highly correlated with certain clinical trait was selected for further analysis.

### Identification of hub genes

Hub genes comprised highly interconnected nodes within a module, and have been shown to be functionally significant [24]. In this study, hub genes were defined as genes with high module membership (MM) (cor.Weighted > 0.8) [25]. We identified hub genes in the module which were highly correlated with certain clinical trait. Furthermore, in the selected module, the protein-protein interaction (PPI) network of the genes was also constructed. The interaction between genes was regarded positive with a combined score of ≥ 0.8 based on the STRING database (http://www.string-db.org/). In the PPI network, genes with a connectivity degree of ≥ 10 were also defined as hub genes. The common hub genes in both co-expression network and PPI network were regarded as “real” hub genes for further analyses.

### Hub gene validation

In the test set of GSE6764, linear regression analyses were conducted to validate the role of hub genes in the progression of HCC. In the training set, the hub genes were extracted for survival and recurrence analyses to identify their roles in HCC prognosis. The RNA-sequencing data were also used to validate the role of hub genes in the prognosis.

### Gene set enrichment analysis (GSEA)

In the RNA-sequencing data, 423 HCC samples were divided into two groups according to the expression level of hub genes respectively. To identify potential function of the hub gene, GSEA (http://software.broadinstitute.org/gsea/index.jsp) [26] was conducted to detect whether a series of priori defined biological processes were enriched in the gene rank derived from DEGs between the two groups. For use with GSEA software, the collection of annotated gene sets of c2.cp.kegg.v5.2.symbols.gmt in Molecular Signatures Database (MSigDB, http://software.broadinstitute.org/gsea/msigdb/index.jsp) was chosen as the reference gene sets. FDR < 0.05 was chosen as the cut-off criteria.

## SUPPLEMENTARY MATERIALS FIGURE AND TABLE


