# Supplementary material for: Co-expression network analysis identified six hub genes in association with metastasis risk and prognosis in hepatocellular carcinoma
Source: Oncotarget. 2017 Apr 6;8(30):48948–58. doi: 10.18632/oncotarget.16896 (PMC5564739; doi:10.18632/oncotarget.16896)
Supplement: Supplementary file 1 [file oncotarget-08-48948-s001.pdf]

Co-expression network analysis identified six hub genes in association with metastasis risk and prognosis in hepatocellular carcinoma

Supplementary Materials

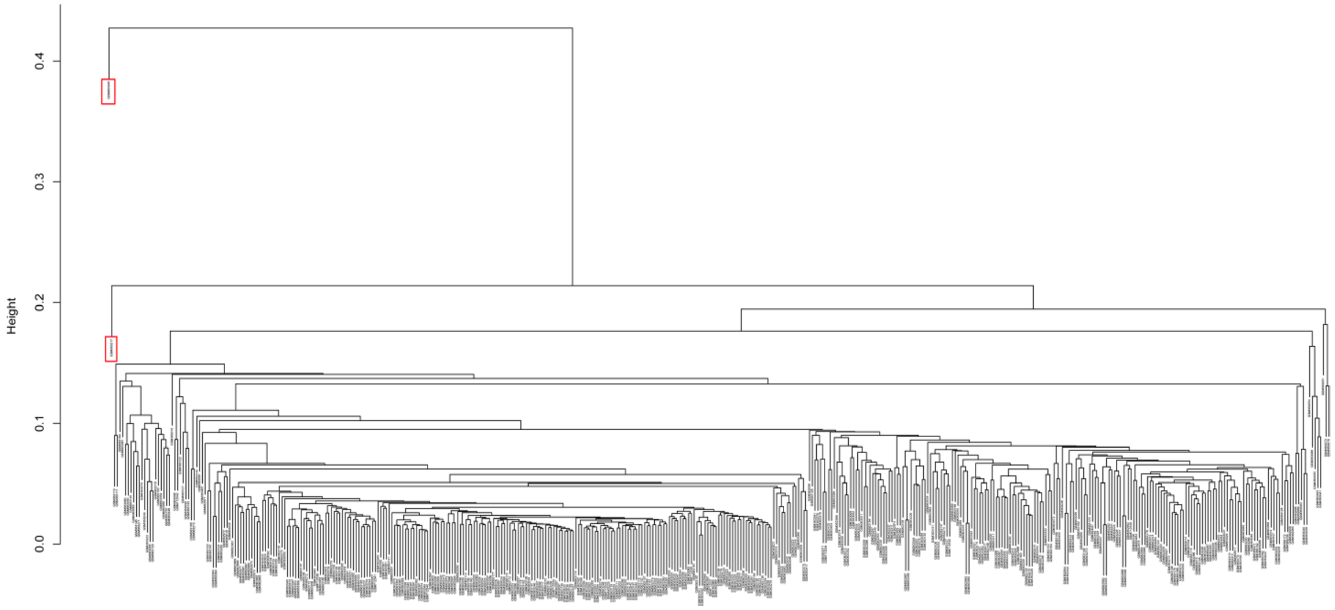

Supplementary Figure 1: Samples clustering to detect outliers. Outliers were marked by red box.

Supplementary Table 1: The results of gene set enrichment analysis (GSEA)

Gene	Gene set name	Size	NOM *p*-val	FDR *q*-val
CYP4A11	KEGG_ARGININE_AND_PROLINE_METABOLISM	54	0.00984252	0.04466988
CYP4A11	KEGG_DRUG_METABOLISM_OTHER_ENZYMES	49	0.007920792	0.045204297
CYP4A11	KEGG_FATTY_ACID_METABOLISM	40	0.002028398	0.04064595
CYP4A11	KEGG_GLYCINE_SERINE_AND_THREONINE_METABOLISM	31	0.011881189	0.044702593
CYP4A11	KEGG_GLYCOLYSIS_GLUCONEOGENESIS	62	0	0.047567338
CYP4A11	KEGG_PROPANOATE_METABOLISM	32	0	0.044567596
CYP4A11	KEGG_RETINOL_METABOLISM	63	0.001996008	0.045562573
DAO	KEGG_VALINE_LEUCINE_AND_ISOLEUCINE_DEGRADATION	44	0.001862197	0.045630652
CYP4A11	KEGG_BETA_ALANINE_METABOLISM	22	0.003921569	0.039482556
EHHADH	KEGG_BETA_ALANINE_METABOLISM	22	0.002114165	0.04532506
ALDH6A1	KEGG_BIOSYNTHESIS_OF_UNSATURATED_FATTY_ACIDS	22	0.007662835	0.048352275
CYP4A11	KEGG_BIOSYNTHESIS_OF_UNSATURATED_FATTY_ACIDS	22	0.006012024	0.047735304
ALDH6A1	KEGG_CITRATE_CYCLE_TCA_CYCLE	30	0.022944551	0.04493482
CYP4A11	KEGG_CITRATE_CYCLE_TCA_CYCLE	30	0.026717557	0.04893512
CYP4A11	KEGG_HISTIDINE_METABOLISM	29	0.006085193	0.045396846
DAO	KEGG_HISTIDINE_METABOLISM	29	0.005639098	0.049265344
CYP4A11	KEGG_INSULIN_SIGNALING_PATHWAY	137	0.002004008	0.045711208
DAO	KEGG_INSULIN_SIGNALING_PATHWAY	137	0	0.04504649
CYP4A11	KEGG_LYSINE_DEGRADATION	44	0	0.030853108
EHHADH	KEGG_LYSINE_DEGRADATION	44	0.006097561	0.042179737
ALDH6A1	KEGG_PPAR_SIGNALING_PATHWAY	69	0.006060606	0.0493711
CYP4A11	KEGG_PPAR_SIGNALING_PATHWAY	69	0.008	0.043602936
CYP4A11	KEGG_SELENOAMINO_ACID_METABOLISM	26	0.008333334	0.043966673
EHHADH	KEGG_SELENOAMINO_ACID_METABOLISM	26	0.024793388	0.04877707
ALDH6A1	KEGG_STARCH_AND_SUCROSE_METABOLISM	49	0	0.04771494
CYP4A11	KEGG_STARCH_AND_SUCROSE_METABOLISM	49	0	0.04236301
ALDH6A1	KEGG_TRYPTOPHAN_METABOLISM	40	0.005882353	0.048187822
CYP4A11	KEGG_TRYPTOPHAN_METABOLISM	40	0.001960784	0.043768384
CYP4A11	KEGG_ALANINE_ASPARTATE_AND_GLUTAMATE_METABOLISM	32	0	0.042099196
DAO	KEGG_ALANINE_ASPARTATE_AND_GLUTAMATE_METABOLISM	32	0.001934236	0.04785948
EHHADH	KEGG_ALANINE_ASPARTATE_AND_GLUTAMATE_METABOLISM	32	0.002096436	0.047264945
CYP4A11	KEGG_COMPLEMENT_AND_COAGULATION_CASCADES	68	0.008492569	0.04539779
DAO	KEGG_COMPLEMENT_AND_COAGULATION_CASCADES	68	0.001869159	0.049896725
EHHADH	KEGG_COMPLEMENT_AND_COAGULATION_CASCADES	68	0.007968128	0.047319017
ALDH6A1	KEGG_CYSTEINE_AND_METHIONINE_METABOLISM	34	0.011673152	0.047433827
CYP4A11	KEGG_CYSTEINE_AND_METHIONINE_METABOLISM	34	0	0.04548778
EHHADH	KEGG_CYSTEINE_AND_METHIONINE_METABOLISM	34	0.010183299	0.04485606
CYP4A11	KEGG_DRUG_METABOLISM_CYTOCHROME_P450	71	0.002004008	0.041317035
DAO	KEGG_DRUG_METABOLISM_CYTOCHROME_P450	71	0	0.047369774
EHHADH	KEGG_DRUG_METABOLISM_CYTOCHROME_P450	71	0.00409836	0.049592838
ALDH6A1	KEGG_PEROXISOME	77	0	0.04407879
CYP4A11	KEGG_PEROXISOME	77	0	0.02629046
DAO	KEGG_PEROXISOME	77	0	0.035167508
ALDH6A1	KEGG_TYROSINE_METABOLISM	42	0	0.045588132
CYP4A11	KEGG_TYROSINE_METABOLISM	42	0.004016064	0.043317467
DAO	KEGG_TYROSINE_METABOLISM	42	0	0.045740474

FDR: false discovery rate.
